# The Grass Carp Genomic Visualization Database (GCGVD): an informational platform for genome biology of grass carp

**DOI:** 10.7150/ijbs.32860

**Published:** 2019-08-07

**Authors:** Min Tang, Ying Lu, Zhongmin Xiong, Ming Chen, Yufang Qin

**Affiliations:** 1 College of Information Technology, Shanghai Ocean University, Shanghai, 201306, China.; 2 Key Laboratory of Fisheries Information Ministry of Agriculture, Shanghai, 201306, China.; 3 College of Fisheries and Life Science, Shanghai Ocean University, Shanghai, 201306, China.

**Keywords:** Grass carp, Database, Conserved domain, RNA-seq

## Abstract

With the release of the draft genome of the grass carp, researches on the grass carp from the genetic level and the further molecular mechanisms of economically valuable physiological behaviors have gained great attention. In this paper, we integrated a large number of genomic, genetic and some other data resources and established a web-based grass carp genomic visualization database (GCGVD). To view these data more effectively, we visualized grass carp and zebrafish gene collinearity and genetic linkage map using Scalable Vector Graphics (SVG) format in the browser, and genomic annotations by JBrowse. Furthermore, we carried out some preliminary study on a whole-genome alternative splicing (AS)of the grass carp. The RNA-seq reads of 15 samples were aligned to the reference genome of the grass carp by Bowtie2 software. RNA-seq reads of each sample and density map of reads were also exhibited in JBrowse. Additionally, we designed a universal grass carp genome annotation data model to improve the retrieval speed and scalability. Compared with the published database GCGD previously, we newly added the visualization of some more genomic annotations, conserved domain and RNA-seq reads aligned to the reference genome. GCGVD can be accessed at http://122.112.216.104.

## Introduction

As one of the four major Chinese carps and an important aquaculture species, the grass carp (*Ctenopharyngodon idella*) is the fresh water economic fish with the largest output in China even in the world. In 2016, the production in China was 5.9 million tons, the highest of all freshwater fish 1, 2. Until now, the research on the grass carp has been involved in many aspects such as health breeding 3, 4, disease prevention 5, 6 and nutrition and feed 7, 8 . The complex biological mechanisms are required to be researched at the whole-gene scale. To integrate and display the whole-genome data for researchers, developing an easy-to-understand, researcher-friendly grass carp genome database is important to support further research.

With the rapid advancement of sequencing technology, the sequencing of a large number of teleosts has been completed, including zebrafish 9, medaka 10, fugu 11, cod 12, tetraodon 13. Correspondingly, the web-based fish genome databases have also been developed. The well-known databases including The Zebrafish Information Network (ZFIN, http://zfin.org/) for zebrafish 14-16, the University of Tokyo Genome Browser Medaka database for medaka 17, the catfish genome database cBARBEL (http://catfishgenome.org) for catfish 18 and SalmonDB (http://genomicasalmones.dim.uchile.cl) for Atlantic salmon and Rainbow trout 19, etc. All of these databases contain genetic, genomic and developmental data of related fishes, while SalmonDB is a multi-organism database. All of them have done plenty of work on gene function annotations, data retrieval and data visualization to facilitate the utilization of genome data. To some extent, these databases promote research on related fish genome. The draft genome of the grass carp was completed by researchers from Institute of Hydrobiology of the Chinese Academy of Sciences, National Genetic Research Center of Chinese Academy of Sciences and Zhongshan University, etc. The project produced a large amount of genomic annotations data resources 20. Chen et al. developed a grass carp genome database (GCGD) to exhibited genome features and annotations of grass carp 21. However, the database is limited by data resources and need to furtherly update. Based on this, we developed a new database to add some more data, especially emphasizing the visualization of these data. Also, we paid an attention to the research of alternative splicing of the grass carp.

In recent years, the rapid development of high-throughput sequencing technology can directly cover most mRNA sequences by directly sequencing mRNA fragments, identify new isoforms produced by splicing, and accurately provide quantitative information. This technology can greatly enhance the understanding and research on the AS of eukaryotes from the molecular level 22. Grass carp is one of the lower vertebrates, the studies on AS of grass carp help to understand the evolution of grass carp genome and immune system, and might contribute to the prevention of disease in aquaculture 23, 24. Although there have been previous studies on the regulation of immune system by alternative splicing in grass carp 25, it is far from enough to study the complex physiological mechanisms of grass carp.

In this paper, we made some efforts on the visualization of the genetic and genomic data of the grass carp. First, we integrated related genetics and genomics data resources of the grass carp, including genome sequence of the female grass carp derived from a five-year-old gynogenetic individual with mature ovary, the gene collinearity between the grass carp and the zebrafish, conserved domains, genetic linkage map which contain 24 linkage groups (LG), several non-coding RNA genes consisting of the miRNA, snRNA, C/D snoRNA, H/ACA snoRNA, rRNA and tRNA, repeat annotation, heterozygous SNPs and the annotations of 27, 263 genes in the female genome 20. Then JBrowse 26 was used to visualize the above data. SVG 27 was also employed in the browser-side to enrich visual form of data. For example, the linkage groups and the gene collinearity between the grass carp and the zebrafish was visualized in this way, so that researchers can better identify known or unknown data patterns, analyze differences and conduct effective data mining. In addition, we focused on the publicly available RNA-Seq data to characterize AS in the grass carp. RNA-seq reads were aligned to the reference genome based on high-throughput sequencing from 15 samples with Bowtie2 28. Again, JBrowse is used to visualize the RNA-seq reads and the density of reads. Finally, we designed a general genome data management model to store the grass carp genome annotation data. With the model, the search speed and scalability of overlapping regions have effectively been improved.

## Materials and methods

### Materials and overview

Related genetic and genomic data resources in the database include the reference genome, the annotations of 27,263 protein-coding genes, 644,817 heterozygous SNPs, the predicted 1, 538 tRNA, 24 rRNA, 207 small nucleolar RNA (snoRNA), 136 small nuclear RNA (snRNA) and 444 microRNA (miRNA) genes of non-coding RNA genes, repeat annotation, conserved functional domains, RNA-seq Reads Per Kilobase per Million (RPKM) of 6 tissues and gene synteny between grass carp and zebrafish. The data above were available for downloading on the official National Center for Gene Research Website (http://www.ncgr.ac.cn/grasscarp/). It can be also downloaded from GCGVD currently. RNA-seq data can be obtained from European Molecular Biology Laboratory (EMBL, https://www.embl.org/) under accession ERS430059.

**Figure 1:**
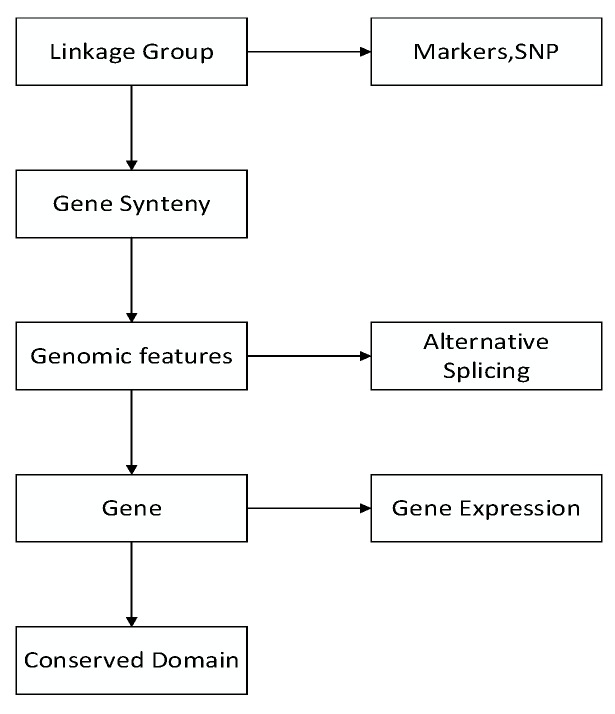
The pipeline of visualizing the genetic and genomic data of the grass carp.

GCGVD was developed in following framework in Figure 1. First we visualized linkage groups, gene synteny and genomic features. Then some other genetic data, e.g., gene expression, conserved domain, genetic markers and SNP, were represented in our database. Meanwhile we aligned RNA-Seq data to the reference genome of the grass carp and visualized them in database.

### Linkage groups

Linkage map is a map of the genes on a chromosome or a scaffold based on linkage analysis. A linkage map shows the relative positions between genes, as determined by how often two gene loci are inherited together. The closer two genes are, the more often they will be inherited together. Based on a genetic linkage map of the grass carp published previously, the 114 scaffolds were anchored on 24 reference genetic linkage groups by 226 genetic markers and 16 SNPs 20. Marker names, SNPs and genetic distances were collected and mapped to 24 linkage groups of the genetic linkage map. We presented them in SVG format on the web interface separately. SVG is a graphical format for describing two-dimensional vector graphics. It is suitable to draw static images, which do not depend on resolution and have high fidelity. And graphical objects can be grouped, styled, transformed and composited into previously rendered objects 27. Therefore the high-quality and non-distorting SVG format is appropriate for genetic linkage groups.

**Figure 2:**
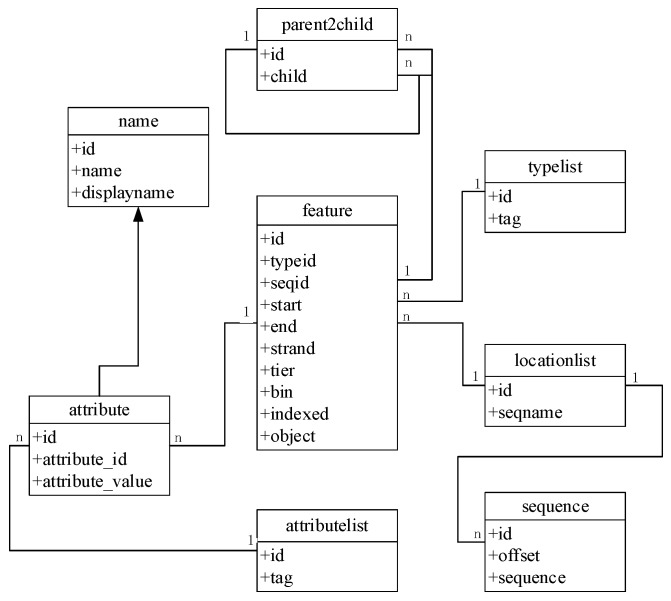
The grass carp genome annotation data model.

To improve the speed and scalability of retrieval in GCGVD, we proposed a grass carp genome annotation data model. The detailed frame is shown in Figure 2. The feature table is the main table, which is responsible for recording information such as the type and starting position of annotations. The reference sequences are stored in the locationlist table and the sequence table at the bottom of Figure 2. And the type of annotations and attribute name are stored in the typelist and attributelist table. Eventually the parent2child table is responsible for concatenating all the information and the tables. Compared with the traditional database relational model, the genome location in our model is indexed according to the hierarchical structure and each genome annotation is assigned to the smallest bin. Hence using this model to store and manage data could accelerate the retrieve and discovery of genomic data.

### Gene collinearity

Both the grass carp and the zebrafish belong to the family *Cyprinidae*. Previous reports indicated that alignment of genes showed high synteny between the two species, and up to 24,018 grass carp genes (88% of the total 27,263 genes) were located on syntenic blocks 20. Visualization of the gene collinearity between the grass carp and the zebrafish may give some implications in studying the evolution of grass carp. In the present paper, we used SVG format to make a synteny diagram between grass carp and zebrafish on web interface in hope that the researchers could find the information more conveniently. The genome of the zebrafish is at the level of chromosomes and the grass carp is at the level of scaffolds, this is because that the grass carp has not yet been assembled to the level of chromosomes. Here we use the SVG format instead of a set of visualization software, including Circos 29 and MCScanX 30, because our visualization method is intuitive and direct for researchers who are interested in the genomes of the grass carp.

### Genome features

Prepared DNA for genome sequencing was extracted the blood sample from a gynogenesis individual 20. 27,263 predicted protein-coding genes were annotated totally. The heterozygous SNPs were detected by SSAHA_Pileup when all of the used paired-end reads were mapped to the assembled scaffolds. Then six thresholds were used to post-filter unreliable SNPs. Finally 644,817 heterozygous SNPs were obtained. Non-coding RNA genes including miRNA, snRNA, C/D snoRNA and H/ACA snoRNA genes were predicted by INFERNAL 31 software against the Rfam database. The rRNA fragments were identified by aligning the rRNA template sequences using Blastn with E-value at 1e-10 and tRNA genes was predicted by the tRNAScan-SE 32 algorithms with default parameters. A *denovo* repeat prediction of grass carp genome was carried out by successively using RepeatModeler (version1.0.5) and RepeatMasker (version 3.3.0).

We visualized the genetic and genomic data above using JBrowse. JBrowse supports files in multiple formats. For example, a FASTA or GFF file for reference sequence, a GFF/BED file for feature data, etc. The available reference sequence file is a file of FASAT format and the gene models file is a file of GFF3 format. Both of them were easily rendered using JBrowse. Several files of non-standard form, e.g., the heterozygous SNPs, non-coding RNA genes and repeat annotation, were converted into GFF3 format. As we know, the GFF3 file contains nine columns of specific information separated by the TAB key. Our operations on text include deletion, substitution, addition, and order adjustment of columns, etc. Several python scripts were used to unify file format into GFF3 for other data file without a suitable format.

### Conserved domain

Conserved domains are recurring units in molecular evolution 33. Gene function can be annotated by prediction of the conserved domains. The prediction can be performed by Interpro 34 in available databases, including ProDom, PRINTS, Pfam, Panther, Profile, PIR, Smart and Pattern. Then the gene functional ontology was retrieved from the outputs of InterPro using Gene Ontology. Here we visualized conserved domains through mapping to the corresponding genes. For each conserved domain, we first compute the relative start site and ending site when mapping to the gene. The algorithm of calculating the sites was executed with a script. Then we eliminated all the rows of low credibility (p-value ≥ 0.05) in the annotations of the conserved domains, and visualized the data with JBrowse.

Additionally, we collected RPKM values of 6 tissues of the grass carp, consisting of Head kidney, Embryo, Liver, Spleen, Brain and Kidney. For each gene in 6 tissues, we generated a histogram of RPKM with a python script and presented it in our database.

### Alternative splicing

In order to study physiological mechanisms of the grass carp more effectively through AS, we collected RNA-seq of 15 samples from the database EMBL. The RNA-seq reads were aligned to the reference genome of the grass carp with Bowtie2 software 28, followed by BAM file sorting with SAMtools 35. According to the start and ending positions of the file obtained, it is easy to get the GFF3 file that contains the start and ending positions information with a python script. RNA-seq reads of the grass carp were visualized by JBrowse. The density diagram of reads for each sample can be obtained with built-in scripts of JBrowse. Fixed bp, e.g., 100bp, 50bp, can be adjusted through the operation on the web interface.

## Results

### Implementation

Several software packages were utilized to construct the database, including the operating system, Ubuntu Kylin 16.04 LTS (http://www.ubuntu.com/); Apache web server 2.4.6 (http://www.apache.org/); MySQL database management system, version 5.5 (http://www.mysql.com/) and PHP, version 5.4.16 (http://php.net/). Python with version 2.7.8 (https://www.python.org/) was used for several scripts and JBrowse package (v1.14.2, http://jbrowse.org/) for displaying the available genome data. HTML5, CSS3 and JQuery were also employed to optimize users' experience.

### Visualizing the linkage groups and gene markers of the grass carp

The linkage maps are meaningful for finding traits of interest and helping researchers to locate other markers. Here we anchored 114 scaffolds on the 24 linkage groups with SVG format, covering 573 Mb (64%) of the female assembly with 17,456 (64%) annotated genes localized. All the 24 linkage groups are shown in the 'Genetic Linkage Map' page. For each linkage group, marker names are on the right side and genetic distances are on the other. Both the search box and the drop-down option frame can help find the marker of interest. When clicking on the marker names or genetic distances, researchers can view the detailed information of the markers from Genbank database.

### Visualizing the gene collinearity between grass carp and zebrafish

**Figure 4:**
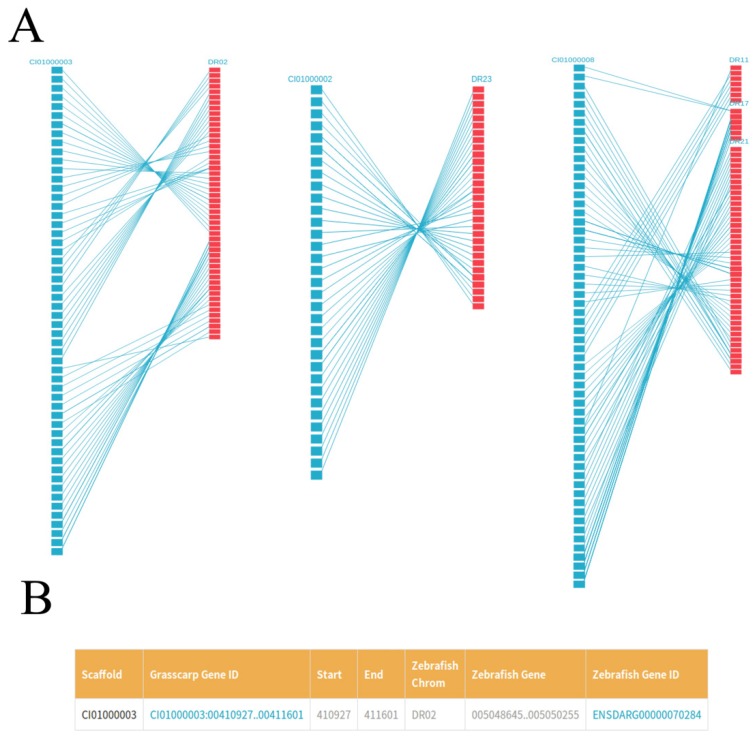
** Gene collinearity between the grass carp and the zebrafish.** A, For each synteny diagram, the grass carp scaffold (left) is represented by blue blocks (for example, CI00000003). Each block represents a grass carp gene. The zebrafish chromosome (right) is represented by red blocks (for example, DR02). Each block represents one or several zebrafish genes. Homologous genes are connected by blue lines. B, Details of some homologous genes between zebrafish and grass carp are presented in a tabular format.

We plotted the gene synteny diagram between each scaffold of the grass carp and homologous zebrafish chromosomes by SVG format so that researchers can observe the relationship between them more intuitively. These results were showed in 'Gene Collinearity' page (Figure 4A). For each synteny diagram, the blue blocks on the left represent all the genes for each scaffold of the grass carp. The red blocks on the right represent the homologous genes of zebrafish and the straight lines connecting the two ends represent the collinearity between the genes of the two species. Genes of the grass carp in synteny diagram are arranged from top to bottom according to their location. By the gene ID, for example, CI01000000_00034855_00045035, we first enter the scaffold ID in the search box, i.e., CI01000000. After that, moving the mouse pointer over the blue block can easily find the specific gene of the grass carp and the corresponding homologous zebrafish genes. Clicking blocks can view the details of the grass carp genes and the corresponding homologous zebrafish genes. More detailed gene information and related information can be seen in JBrowse by clicking on 'Grass carp Gene ID' (Figure 4B). Similarly, clicking on 'Zebrafish Gene ID' could get more information about zebrafish genes from EMBL.

### Visualizing the genome information of the grass carp

**Figure 5:**
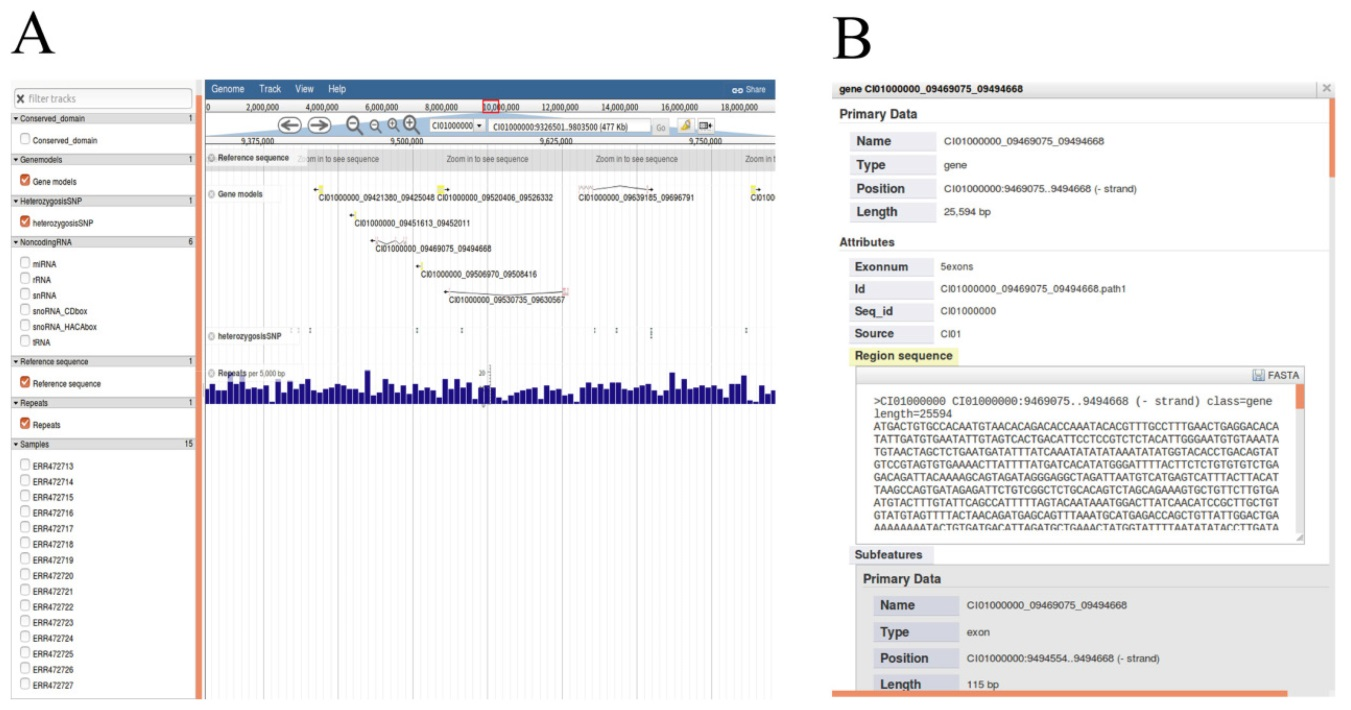
** All available data of genome annotations rendered in JBrowse**. A, Selected data appears graphically on the right main window by selecting the options in the left frame. B, Detailed annotation of protein-coding gene will be displayed after clicking on the gene structure. Detailed annotation of other data can also be rendered selectively as described operation above.

We chose JBrowse, an interactive genome visualization browser to display genetic and genomic data resources including genome sequence, annotations of protein-coding genes, heterozygous SNPs, non-coding RNA genes and repeat annotation (Figure 5A). Researchers can enter the 'JBrowse' page to view the data of interest by clicking the check buttons to select the dataset tracks and biological characteristics listed in the left panel. All selected tracks are displayed in the main window, and the track is dynamic, supporting operations such as zooming in, zooming out and pan. The search box in the upper function bar supports retrieval of locations of all scaffolds and help researchers quickly locates regions of interest. Detailed annotations can be obtained by clicking units of selected tracks in the main window. As an example, detailed gene annotations will appear by clicking gene structure after checking 'Gene models' button (Figure 5B).

### Mapping conserved domains to the genes

**Figure 6:**
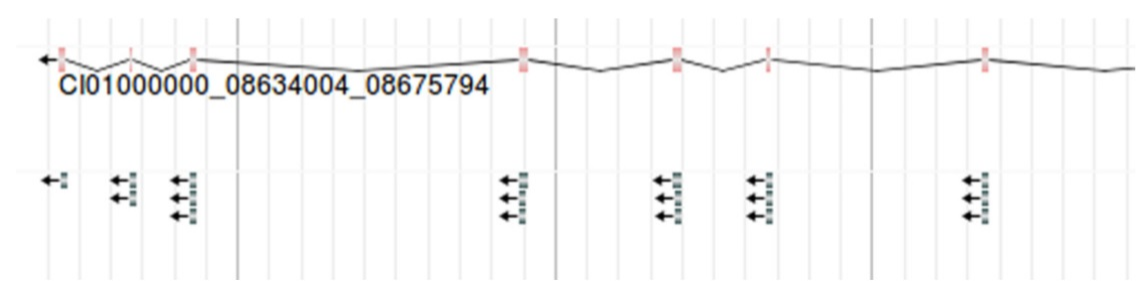
** The conserved domain under a gene.** Each line with arrow represents a conserved domain. For example, CI00000000:08634004. 08675794 is shown.

As the method mentioned above, we successfully mapped the conserved domains to the exons of the corresponding gene, and then visualized the conserved domains encoded by the gene in JBrowse. The gene structure is in the upper of the figure, followed by the conserved domains encoded by its exon at the bottom of the figure (Figure 6). Each domain is displayed through different lines. Moving your mouse over the domain will display its corresponding domain information including InterPro Entry, Domain Name, etc. The retrieved Gene Ontology (GO) terms are also included.

**Figure 7:**
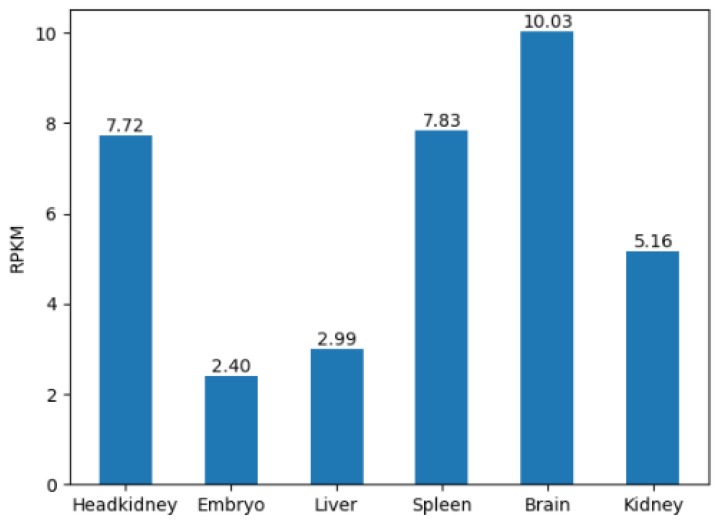
** The gene expression in 6 tissues.** For example**,** RPKM of gene CI01000000_00034855_00045035 is shown.

In addition, we considered the expression levels of six tissues in the grass carp. For any gene in 6 tissues of the grass carp, the RPKM were presented in the form of a histogram. Visualizing the gene expression of the grass carp allows researchers to assess the level of expression of a target gene intuitively (Figure 7). RPKM of 6 tissues are displayed on the 'Expression' page. When retrieving the RPKM of one gene, we just enter the gene ID in the search box, for example, CI01000000_00034855_00045035.

### Aligning RNA-seq reads to the reference genome

**Figure 8:**
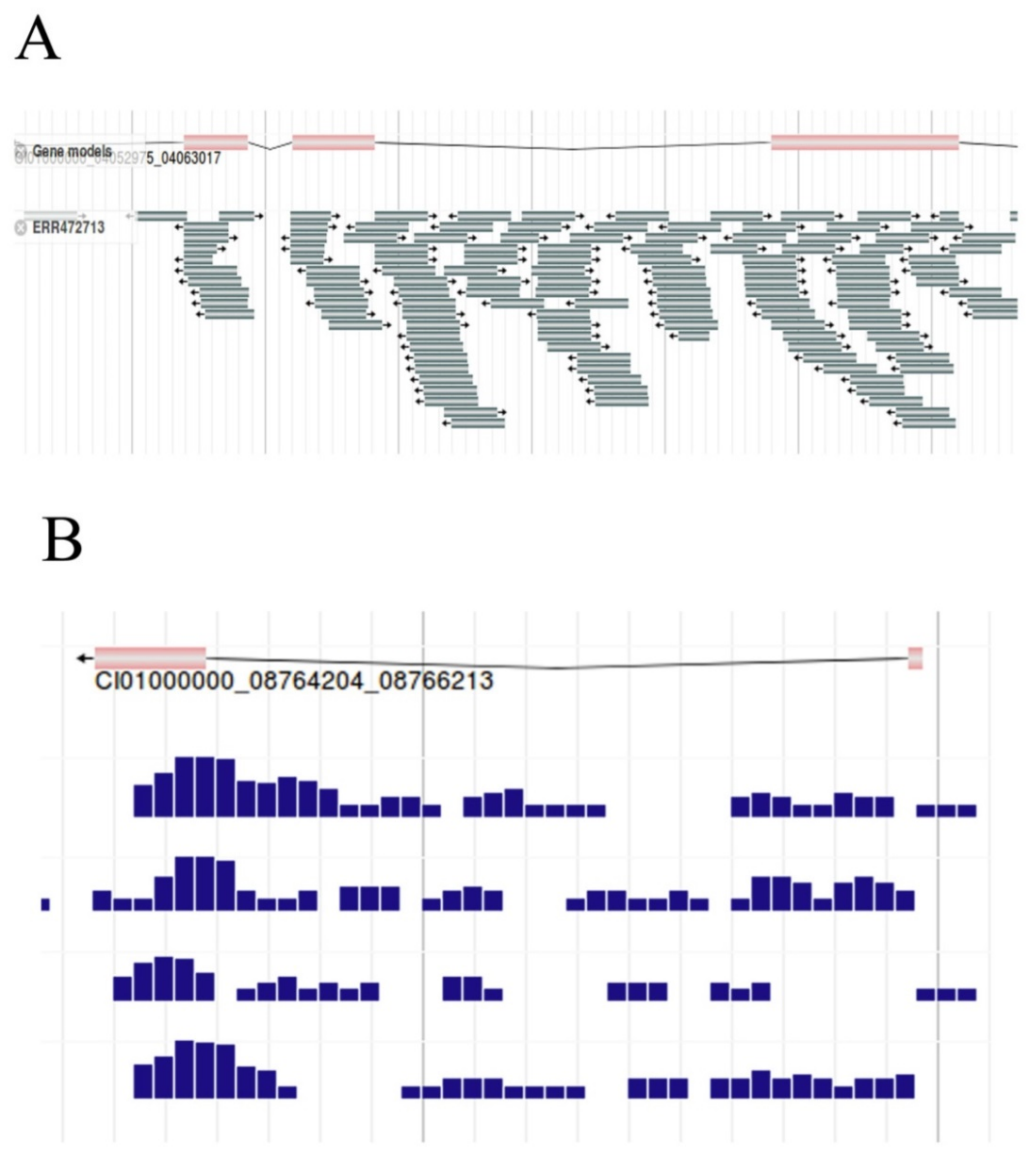
** RNA-seq reads of grass carp.** A, RNA-seq reads (Sample id 'ERR472713') were mapped to the reference genome. B, Density map of reads per 50bp. Here we captured the density map of 4 samples.

As the basic work of AS research, the filtered RNA-seq reads were aligned against the reference genome of the grass carp by Bowtie2, here we visualized the results with JBrowse. Gene structure was shown in the upper of the figure, and the reads aligned against the reference genome below (Figure 8A). By narrowing the canvas to a certain extent by the zoom out button on the function bar, the density of reads for each sample can be obtained under fixed bp (Figure 8B). For example, samples can be selected to present the density of reads per 100bp, per 50bp, etc. With the assisting of the density map, differences in the density of reads can be clearly observed, which could help to diagnose genes that might be differentially expressed to a certain degree.

## Discussion

In this paper, we established a web-based grass carp genomic visualization database. Linkage groups and gene synteny are drawn by SVG, which is more effective for high-fidelity documents. On the other hand, using SVG gives us the flexibility to customize the visual form of our data to enhance data readability. The visualized images of gene expression are drawn by the script and then stored in the database as images. And other visualizations consisting of genome features, conserved domain and RNA-seq reads are done with JBrowse. Compared with the previously grass carp genome database (GCGD) 21, in genomic information display window, we newly added the annotations of heterozygous SNPs, non-coding RNA genes and repeats annotation, etc. Additionally, we mapped the conserved domains to the corresponding gene and visualize them. We also did some preliminary work on the alternative splicing research. The RNA-seq of 15 samples was mapped to the reference sequence by alignment, and these results were displayed by JBrowse. Finally, we established the underlying database based on the grass carp genome annotation data model. The detailed comparisons are shown in Table 1.

It is worth emphasizing that our database focuses on the visualization of the genetic and genomic data of the grass carp. Although the visualization system of the genome annotation data has a considerable development, for example, UCSC Genome Browser 36, NCBI MapViewer 37 and EBI Ensembl 38, they provide browsing, retrieval and visualization of genome annotation data under a relatively efficient database. As a general and open genome browser, our database offers the visualization of various genome annotation data, especially supplemented the data visualization that cannot be presented in track format.

Due to the limited genetic and genomic data available at present, our work focuses on the annotations of genome and the visualization of related data. With further researching on the grass carp genome, the gene annotations in the database will be continuously updated. Other omics data include further studies on alternative splicing will be gradually introduced into the database. In the future we hope that the database would become a highly understood genomic resource platform and data mining work bench of grass carp.

## Figures and Tables

**Figure 3:**
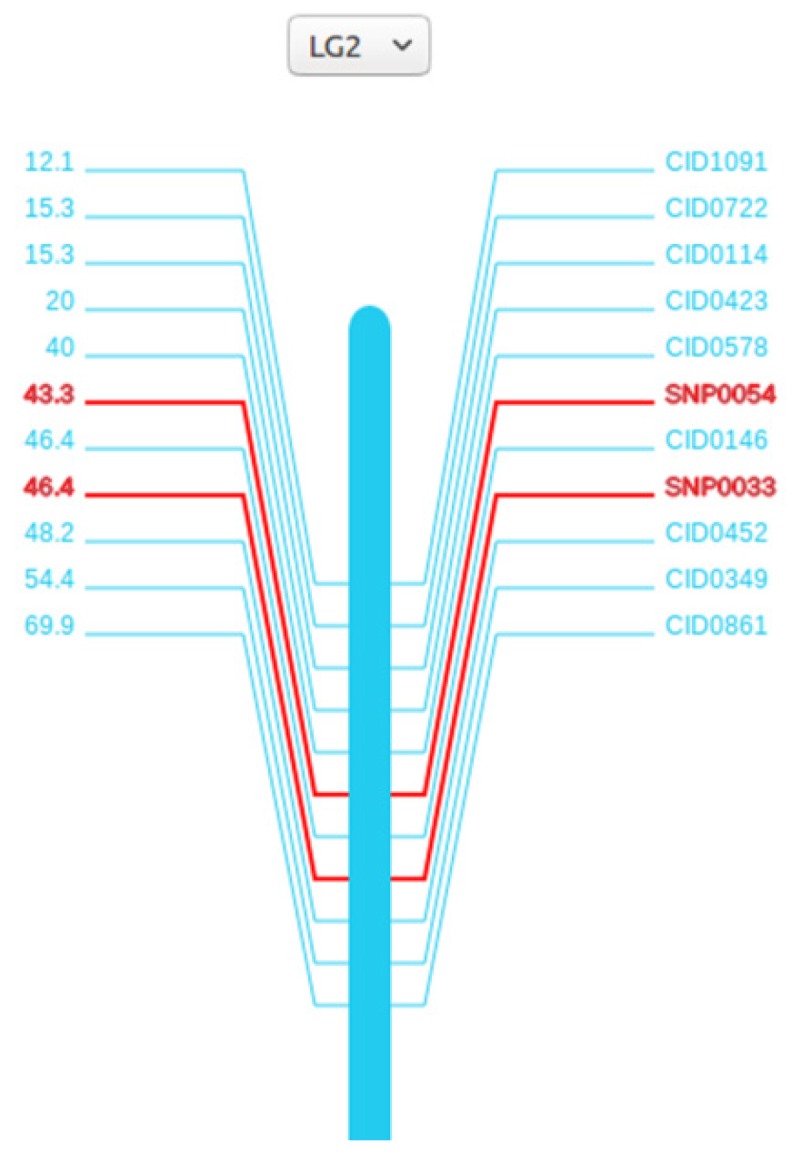
** Genetic map of the second linkage group (LG2)**. A vertical bar is in the middle, marker names and genetic distances are on the sides. For SNPs, we specially marked red to distinguish it with others.

**Table 1:** The comparison of the two grass carp genome databases.

Comparative aspects	GCGVD	GCGD	The advantage of GCGVD
The main data in the database	Functional annotations, conserved domain, SNP, non-coding RNA genes, repeat annotation, RPKM of six tissues, gene collinearity, genetic map, RNA-seq reads, etc.	Functional annotations, gene collinearity, genetic map, SSRs, three transcriptomic datasets, etc.	GCGVD added some annotation data not available in GCGD.
Data management and storage	Designed a general annotation data model to store and manage data.	Ordinary storage.	GCGVD has higher retrieval speed and scalability in overlapping regions.
